# Cathepsin D regulates cerebral Aβ42/40 ratios via differential degradation of Aβ42 and Aβ40

**DOI:** 10.1186/s13195-020-00649-8

**Published:** 2020-07-06

**Authors:** Caitlin N. Suire, Samer O. Abdul-Hay, Tomoko Sahara, Dongcheul Kang, Monica K. Brizuela, Paul Saftig, Dennis W. Dickson, Terrone L. Rosenberry, Malcolm A. Leissring

**Affiliations:** 1 Institute for Memory Impairments and Neurological Disorders, University of California, Irvine, Irvine, CA 92697 USA; 2 Department of Neurobiology and Behavior, University of California, Irvine, Irvine, CA 92697 USA; 3 Department of Neuroscience, Mayo Clinic Florida, Jacksonville, FL 32224 USA; 4 Institut für Biochemie, Christian-Albrechts-Universität zu Kiel, 24098 Kiel, Germany

**Keywords:** Alzheimer disease, Amyloid-β protein, Cathepsin D, Proteostasis, Lysosomes

## Abstract

**Background:**

Cathepsin D (CatD) is a lysosomal protease that degrades both the amyloid β-protein (Aβ) and the microtubule-associated protein, tau, and has been genetically linked to late-onset Alzheimer disease (AD). Here, we sought to examine the consequences of genetic deletion of CatD on Aβ proteostasis in vivo and to more completely characterize the degradation of Aβ42 and Aβ40 by CatD.

**Methods:**

We quantified Aβ degradation rates and levels of endogenous Aβ42 and Aβ40 in the brains of CatD-null (CatD-KO), heterozygous null (CatD-HET), and wild-type (WT) control mice. CatD-KO mice die by ~ 4 weeks of age, so tissues from younger mice, as well as embryonic neuronal cultures, were investigated. Enzymological assays and surface plasmon resonance were employed to quantify the kinetic parameters (*K*_M_, *k*_cat_) of CatD-mediated degradation of monomeric human Aβ42 vs. Aβ40, and the degradation of aggregated Aβ42 species was also characterized. Competitive inhibition assays were used to interrogate the relative inhibition of full-length human and mouse Aβ42 and Aβ40, as well as corresponding p3 fragments.

**Results:**

Genetic deletion of CatD resulted in 3- to 4-fold increases in insoluble, endogenous cerebral Aβ42 and Aβ40, exceeding the increases produced by deletion of an insulin-degrading enzyme, neprilysin or both, together with readily detectable intralysosomal deposits of endogenous Aβ42—all by 3 weeks of age. Quite significantly, CatD-KO mice exhibited ~ 30% increases in Aβ42/40 ratios, comparable to those induced by presenilin mutations. Mechanistically, the perturbed Aβ42/40 ratios were attributable to pronounced differences in the kinetics of degradation of Aβ42 vis-à-vis Aβ40. Specifically, Aβ42 shows a low-nanomolar affinity for CatD, along with an exceptionally slow turnover rate that, together, renders Aβ42 a highly potent competitive inhibitor of CatD. Notably, the marked differences in the processing of Aβ42 vs. Aβ40 also extend to p3 fragments ending at positions 42 vs. 40.

**Conclusions:**

Our findings identify CatD as the principal intracellular Aβ-degrading protease identified to date, one that regulates Aβ42/40 ratios via differential degradation of Aβ42 vs. Aβ40. The finding that Aβ42 is a potent competitive inhibitor of CatD suggests a possible mechanistic link between elevations in Aβ42 and downstream pathological sequelae in AD.

## Introduction

Extracellular deposition of the amyloid β-protein (Aβ) is the most widely accepted pathognomonic marker of Alzheimer disease (AD). However, another early and invariant feature of AD is lysosomal dysfunction, and accruing evidence suggests that the lysosome may be a pivotal locus for the molecular pathogenesis of the disease [1, 2]. Aβ is generated in the endolysosomal system by acidic proteases and secreted into the extracellular space, but an as yet unquantified portion is also shuttled to lysosomes [3]. Secreted Aβ is likewise trafficked to lysosomes in an ApoE-dependent manner [4]. More recently, accruing evidence suggests that tau, particularly misfolded variants, is also trafficked to the lysosome via chaperone-mediated autophagy [5]. Misfolded tau, in turn, is widely accepted as the proximal cause of neuronal cell loss and consequent cognitive disturbances in AD and multiple other neurodegenerative diseases [6]. Collectively, these observations suggest that lysosomal disturbances may be highly relevant to the pathogenic role of Aβ and tau and, potentially, their interrelationship.

Cathepsin D (CatD) is a lysosomal aspartyl protease that degrades both Aβ [7, 8] and tau [9] in vitro and is strongly implicated in the pathogenesis of AD and multiple other neurodegenerative diseases [10]. Loss-of-function mutations in CatD result in multiple forms of neurodegeneration in humans [11] and sheep [12]. Moreover, a common variation in the CatD gene (*CTSD*) has been linked to risk for late-onset AD [13] and to elevated levels of both Aβ42 and tau in cerebrospinal fluid [14, 15].

Multiple lines of evidence suggest that impaired Aβ degradation may play a role in the pathogenesis of AD [16, 17]. Several specific Aβ-degrading proteases (AβDPs) have been identified that, when deleted in vivo, result in significant increases in cerebral Aβ levels, including neprilysin (NEP) [18–20], insulin-degrading enzyme (IDE) [21, 22], and many others [16, 17]. Conversely, overexpression of several AβDPs has been shown to dramatically reduce AD-type pathology in mouse models of the disease [23, 24]. Nevertheless, some proteases shown to degrade Aβ in vitro, including CatD, have not yet been thoroughly assessed in vivo.

This study sought to elucidate the role of CatD in Aβ proteostasis in vivo, using CatD-null (CatD-KO) mice and several complementary approaches. CatD-KO mice die prematurely by ~ 4 weeks of age due to peripheral causes and are a well-established model of neuronal ceroid lipofuscinosis [25], but they remain healthy and comparable in body weight to WT mice until ~ 23 days of age [26, 27]. Using tissue extracts from younger CatD-KO mice, cultured embryonic neurons, ELISA measurements in mice across a range of ages (15 to 26 days old), and extensive in vitro experiments, we provide compelling evidence that CatD plays a significant role in Aβ proteostasis in vivo. Although the premature lethality in these mice precludes the assessment of CatD deletion on all aspects of AD-type pathology, our findings suggest that future work on the role of CatD in Aβ proteostasis, using more sophisticated methods for manipulating CatD in a regulatable manner in vivo, is highly warranted.

## Results

### CatD is the major soluble Aβ-degrading protease at acidic pH

**Fig. 1:**
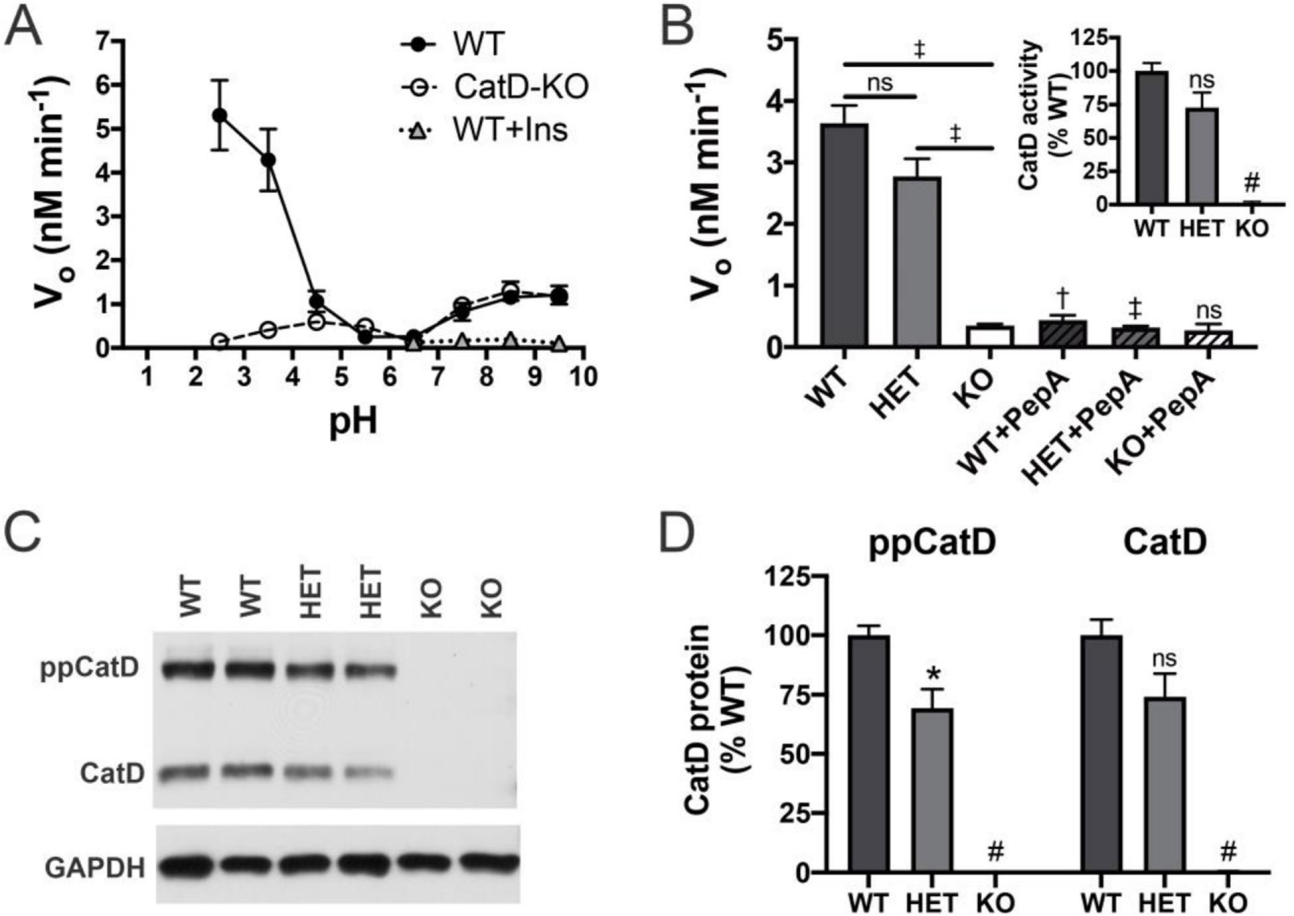
CatD activity and protein levels in brain extracts. **a** Aβ degradation in soluble brain extracts from 15-day-old WT and CatD-KO mice as a function of pH. Note that the abundant Aβ-degrading activity occurring at acidic pH is essentially absent in CatD-KO extracts. Note also that the smaller peak at neutral pH is inhibited by insulin (Ins), reflecting IDE activity [28]. Data are mean ± SEM for 5 replicates. ^†^*p* < 0.01. **b** Aβ-degrading activity in extracts from 15-day-old CatD-KO, CatD-HET, and CatD-WT mice at pH 4.0. Note that the activity in WT and CatD-HET extracts is largely inhibited by the CatD inhibitor, pepstatin A (PepA). Data are mean ± SEM for 4 replicates. ^†^*p* < 0.01; ^‡^*p* < 0.001; ^#^*p* < 0.0001. Inset: CatD activity in brain extracts from WT, CatD-HET, and CatD-KO mice measured directly using a selective substrate. Data are mean ± SEM for 4 replicates.; ^#^*p* < 0.0001. Note also that Aβ-degrading activity in the CatD-HET extracts is not reduced by 50% as expected from deletion of one of two *CTSD* alleles. **c**, **d** Representative western blot (**c**) and quantification of multiple samples (**d**) showing relative CatD levels in CatD-KO, CatD-HET, and CatD-WT mice. Note that, consistent with the activity data in **b**, CatD levels in CatD-HET brains are not 50% of those in WT brains. Data in **d** are mean ± SEM for 6 samples per genotype. *p < 0.05; ^#^*p* < 0.0001

As an initial step towards elucidating the role of CatD in Aβ proteostasis, we quantified rates of Aβ degradation in vitro in soluble brain extracts from CatD-KO mice and wild-type controls [26] as a function of pH, focusing on extracts from 15-day-old mice, due to the premature lethality of CatD-KO mice that occurs by ~ 4 weeks of age [26, 27]. Consistent with previous results [7, 28], Aβ-degrading activity was present principally within two pH ranges: at neutral pH (pH 7.5 to 9.5) and—to a considerably larger extent—also at acidic pH (pH 2.5 to 4.5) (Fig. 1a). The Aβ-degrading activity at neutral pH was inhibited by excess insulin and reflects the activity of the neutral protease insulin-degrading enzyme (IDE), as shown previously by McDermott and Gibson [28]. By contrast, the abundant Aβ-degrading activity at acidic pH in WT brain extracts was essentially absent in extracts from CatD-KO mice, strongly suggesting that CatD is the primary soluble AβDP in the brain (Fig. 1a). To extend and confirm these findings, we quantified Aβ degradation at pH 4.0 in soluble brain extracts from CatD-KO and WT mice, as well as heterozygous null (CatD-HET) mice (Fig. 1b). As expected, the Aβ-degrading activity present in WT (and CatD-HET) extracts at acidic pH was inhibited almost completely by pepstatin A (PepA), a potent CatD inhibitor (Fig. 1b), reinforcing the conclusion that CatD is indeed the principal AβDP operative at acidic pH and ruling out alternative explanations such as compensatory changes in other AβDPs. Confirming this, western blotting revealed that levels of the amyloid precursor protein (APP), APP C-terminal fragments, and two other major AβDPs—IDE and NEP—were unchanged in CatD-KO brains relative to WT controls (Supp. Fig. S1). Surprisingly, however, in CatD-HET extracts, Aβ-degrading activity (Fig. 1b) and CatD activity assessed by a selective substrate (Fig. 1b, inset) were not reduced to 50% of WT levels, as expected, but instead were reduced by considerably less (23.2 ± 10.7% and 26.4 ± 13.4%, respectively, for the two different activity assays; *p* > 0.05 in both cases), suggesting that some degree of compensatory upregulation of CatD occurs in the heterozygous state. Consistent with this, levels of both preprocathepsin D and mature CatD protein in the CatD-HET animals were also determined to be > 50% of WT levels (Fig. 1c, d), with mature CatD protein being reduced by only 26.0 ± 14.2% relative to WT controls (*p* > 0.05; Fig. 1d).

### Deletion of CatD increases insoluble Aβ42 and Aβ40 as well as Aβ42/40 ratios

**Fig. 2:**
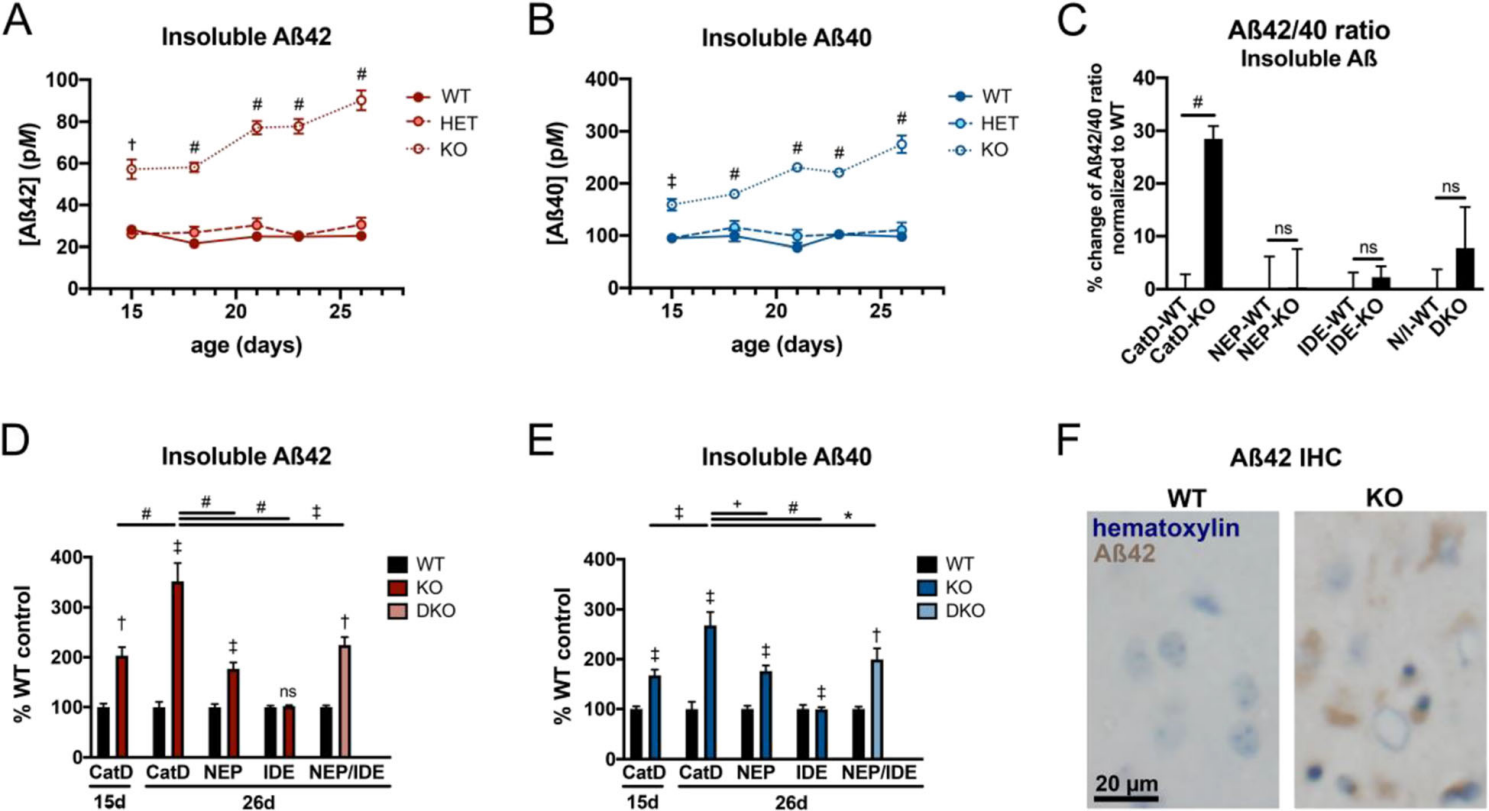
Insoluble Aβ42 and Aβ40 levels in CatD-KO, CatD-HET, and CatD-WT brains. **a**, **b** Levels of insoluble, endogenous brain Aβ42 (**a**) and Aβ40 (**b**) in CatD-KO, CatD-HET, and CatD-WT mice as a function of age. Note that levels of both Aβ species are markedly increased in CatD-KO, but not in CatD-HET, mice relative to WT controls at all ages examined. Data are mean ± SEM for 4–6 replicates per group. ^†^*p* < 0.01; ^‡^*p* < 0.001; ^#^*p* < 0.0001. **c** Insoluble Aβ42/40 ratios are significantly increased in CatD-KO mice, but not NEP-KO, IDE-KO, or NEP/IDE-DKO mice, relative to their respective WT controls. Data are mean ± SEM for 28–30 replicates per group for CatD-KO and CatD-WT mice and 6–11 replicates per group for the other genotypes. ^#^*p* < 0.0001. **d**, **e** Percent increases in insoluble, endogenous brain Aβ42 (**d**) and Aβ40 (**e**) in 15-day-old and 26-day-old CatD-KO mice as compared to 26-day-old NEP-KO, IDE-KO, and NEP/IDE double-knockout (DKO) mice, all normalized to respective WT controls. Note that 26-day-old CatD-KO mice exhibit significantly higher increases in insoluble Aβ42 and Aβ40 above their WT controls than age-matched mice lacking NEP, IDE, or both NEP and IDE. Data are mean ± SEM for 4–6 replicates per group. *p < 0.05; ^†^*p* < 0.01; ^‡^*p* < 0.001; ^#^*p* < 0.0001. **f** Intracellular endogenous Aβ42 accumulation occurs in CatD-KO mice by 3 weeks of age. Shown is immunohistochemical staining of a 26-day-old CatD-KO mouse and age-matched WT control with an anti-Aβ42 end-specific antibody [31]. Additional immunohistochemical characterization is provided in Supp. Fig. S4

To investigate whether CatD regulates cerebral Aβ levels in vivo, we quantified endogenous Aβ levels in the brains of CatD-KO, CatD-HET mice, and WT controls, analyzing both diethylamine (DEA)-soluble and diethylamine-insoluble (guanidinium extracted) cerebral extracts using well-established Aβ42- and Aβ40-specific ELISAs [29–31]. Soluble Aβ is generally believed to reflect primarily monomeric Aβ species, with insoluble Aβ reflecting aggregated forms [31]. Because CatD-KO mice suffer from premature lethality by ~ 4 weeks of age [26, 27], we elected to analyze mice across a range of ages (15 to 26 days old). Relative to age-matched WT controls, levels of insoluble cerebral Aβ42 (Fig. 2a) and Aβ40 (Fig. 2b) were significantly increased in CatD-KO brains at all ages examined, including multiple time points well before any signs of moribundity (which first occurs at ~ 23 days of age [26, 27]). In CatD-KO mice, the concentrations of both peptides rose in an age-dependent manner, culminating in a ~ 4-fold increase in insoluble Aβ42 and a ~ 2.5-fold increase in insoluble Aβ40 in CatD-KO mice relative to WT mice at 26 days of age (Fig. 2a, b). In marked contrast, insoluble Aβ42 and Aβ40 levels in CatD-HET mice were not significantly different from WT controls. The increases in insoluble Aβ42 and Aβ40 in CatD-KO relative to WT controls were highly significant at all ages, both in terms of pairwise comparisons between age-matched groups (Fig. 2a, b) and when analyzed by ANOVA using a mixed-effects model (*p* < 0.0001 for age, genotype, and age × genotype for both Aβ42 and Aβ40).

Of special interest, the percent increase in Aβ42 seemed consistently higher than that of Aβ40 at all ages examined, so we calculated the ratios of insoluble Aβ42 to Aβ40 for all mice examined. Overall, CatD-KO mice showed a highly statistically significant (*p* < 0.0001) ~ 30% increase in insoluble Aβ42/40 ratios relative to WT controls (Fig. 2c), an increase comparable in scale to that induced by many AD-linked presenilin mutations [29, 32, 33]. In contrast, cerebral Aβ42/40 ratios were not significantly changed in mice lacking NEP (NEP-KO) or IDE (IDE-KO)—or both NEP and IDE, simultaneously (NEP/IDE-DKO; Fig. 2c). In parallel, using the same methods, we also quantified insoluble Aβ42 and Aβ40 levels in age- and sex-matched NEP-KO, IDE-KO, and NEP/IDE-DKO mice. Relative to their respective WT controls, the percent increases in insoluble Aβ42 (Fig. 2d) and Aβ40 (Fig. 2e) in 26-day-old CatD-KO mice were found to be significantly higher than those in age-matched NEP-KO, IDE-KO, and NEP/IDE-DKO mice, suggesting that the contribution of CatD to overall brain Aβ proteostasis in vivo exceeds that of both NEP and IDE.

In contrast to the consistently large increases in insoluble—likely aggregated—forms of Aβ seen in CatD-KO mice, levels of endogenous soluble Aβ42 (Supp. Fig. S2A) and Aβ40 (Supp. Fig. S2B) were lower overall, and consequently more variable, but nevertheless exhibited highly significant trends towards decreasing levels as a function of increasing age (*p* < 0.0001 for age × genotype for both Aβ42 and Aβ40 using a mixed-effects multiple comparison ANOVA), with significant decreases in both peptides relative to WT mice evident at 26 days of age (Supp. Fig. S2A,B). Similarly, opposite to the case for insoluble Aβ, soluble Aβ42/40 ratios were significantly decreased in CatD-KO mice relative to WT controls (Supp. Fig. S2C). Nevertheless, because significantly less soluble vs. insoluble Aβ was extracted, the overall (soluble plus insoluble) Aβ42/40 ratios remained significantly elevated in CatD-KO mice (Supp. Fig. S2D). NEP-KO, IDE-KO, and NEP/IDE-DKO mice, by contrast, showed no significant changes in soluble or overall Aβ42/40 ratios (Supp. Fig. S2C,D). Unlike 26-day-old CatD-KO mice, which exhibited lower soluble Aβ levels relative to their WT controls, age-matched NEP-KO and IDE-KO mice showed significant increases in both soluble Aβ42 (Supp. Fig. S2E) and Aβ40 (Supp. Fig. S2F) relative to their respective WT controls.

The fact that CatD-KO mice die at such an early age raises the obvious concern that the elevated Aβ levels may represent a non-specific consequence, rather than a true reflection of the contribution of CatD to brain Aβ proteostasis. Towards the goal of addressing this concern, we quantified cerebral Aβ levels in another mouse model featuring both lysosomal dysfunction and premature lethality: the twitcher mouse. The twitcher mouse harbors a mutation in the galactosylceramidase gene (*GALC*), making it a model of human globoid cell leukodystrophy (Krabbe disease), a lethal lysosomal storage disorder [34, 35]. Depending on the genetic background, twitcher mice die anywhere from 40 days of age to 3 months of age [34, 36], and in our colony, 50% died at ~ 81 days of age. To assess whether Aβ accumulated in this mouse model, we quantified cerebral Aβ levels in CatD-KO and twitcher mice, both prior to the onset of visible neurological symptoms (15 days) and 1–2 days prior to the typical date of death for each model (26 days for CatD-KO; 80 days for twitcher mice). As in previous experiments, relative to WT littermate controls, CatD-KO mice exhibited statistically significant increases in insoluble Aβ42 and Aβ40 (Supp. Fig. S3A, B) and significant decreases in soluble Aβ42 and Aβ40 (Supp. Fig. S3C, D) at 26, but not 15, days of age. In marked contrast, twitcher mice showed no significant increase in soluble or insoluble Aβ42 or Aβ40 at any age tested relative to age- and sex-matched, colony-specific WT controls (Supp. Fig. S3A-D). While the twitcher mouse model is not a perfect control for the specific phenotype in CatD-KO mice, these results lend support to the conclusion that CatD is a bona fide regulator of Aβ proteostasis in vivo.

### CatD-KO mice develop intralysosomal Aβ42 deposits by 3 weeks of age

The preceding ELISA-based results reflect the levels of Aβ averaged over the entire volume of the cerebrum. The ~ 4-fold increase in whole-brain Aβ42 levels induced by the deletion of CatD, however, might theoretically reflect a considerably larger, localized increase in Aβ42 if limited exclusively to lysosomes. Consistent with this prediction, intracellular deposits of endogenous Aβ42 could be readily detected in the brains of 3-week-old CatD-KO mice, but not WT mice, by conventional immunohistochemical methods (Fig. 2f; Supp. Fig. S4A-F). Co-labeling experiments confirmed the presence of abundant Aβ42 in Lamp2-positive lysosomes, which was particularly prominent in neuronal cell bodies in cortical layers III and IV (Supp. Fig. S4G) and in hippocampal CA1 pyramidal neurons (Supp. Fig. S4H). Although Aβ42 is not the only protein expected to accumulate following deletion of CatD, it is notable that neurons containing abundant Aβ42 were also positive for several immunohistochemical markers of amyloid accumulation, including Congo Red, Thioflavin S, and Gallyas silver stains (Supp. Fig. S4I-L).

### Primary neurons lacking CatD show defects in intracellular Aβ catabolism

As an independent method of investigating the role of CatD in Aβ degradation, we studied cultured primary hippocampal neurons obtained from embryonic (E18) CatD-KO and WT littermate mice. Consistent with our in vivo results, significantly more Aβ42 (Supp. Fig. S5A) was secreted into the conditioned medium of CatD-KO neurons relative to WT controls, with a similar, albeit statistically non-significant trend obtained for Aβ40 (Supp. Fig. S5B). To explore whether the observed changes in extracellular Aβ reflected differences in the intracellular catabolism per se, as opposed to the possible effects on Aβ production or secretion, cultured neurons were incubated in the presence of fluorescently labeled Aβ42 and Aβ40, washed to remove excess extracellular Aβ peptides, then allowed to catabolize internalized Aβ for 2 h prior to microscopic analysis. CatD-KO neurons exhibited substantial defects in the catabolism of Aβ42 in particular, and to a lesser extent Aβ40, as determined from the relative amounts of fluorescently tagged Aβ peptides present after the 2-h incubation period (Supp. Fig. S5C-E). Taken together with the findings above, these results strongly suggest that CatD is a powerful regulator of intralysosomal Aβ catabolism, independent of any deleterious phenotype triggered by CatD deletion in vivo.

### Mechanistic basis for the increase in Aβ42/40 ratios

Given that deletion of CatD produced a highly consistent increase in insoluble (and total) cerebral Aβ42/40 ratios, and in light of differential effects of CatD on Aβ42 vs. Aβ40 levels seen in cultured neurons, we focused our attention on the possible mechanisms to account for these seemingly selective effects. Mechanisms affecting the production of Aβ seemed unlikely, based on previous studies demonstrating that Aβ production is unperturbed in CatD-KO neurons [37], as well as our own data (e.g., Supp. Fig. S1). CatD might alternatively affect Aβ42 levels through the conversion of Aβ42 to Aβ40 or other shorter species through carboxypeptidase activity, as has been shown previously for cathepsin B [38]. To explore this possibility, we used mass spectrometry to determine the cleavage sites within human Aβ42 and Aβ40 induced by purified human CatD. Consistent with previous studies [7, 8], CatD hydrolyzed both Aβ40 and Aβ42 at the Phe^19^-Phe^20^ and Phe^20^-Ala^21^ peptide bonds (Supp. Table S1; Supp. Figs. S6, S7). A third cleavage site, which proved to be the major one, occurred at the Leu^34^-Met^35^ peptide bond (Supp. Table S1; Supp. Figs. S6, S7). However, we found no evidence for the conversion of Aβ42 to Aβ40.

**Fig. 3:**
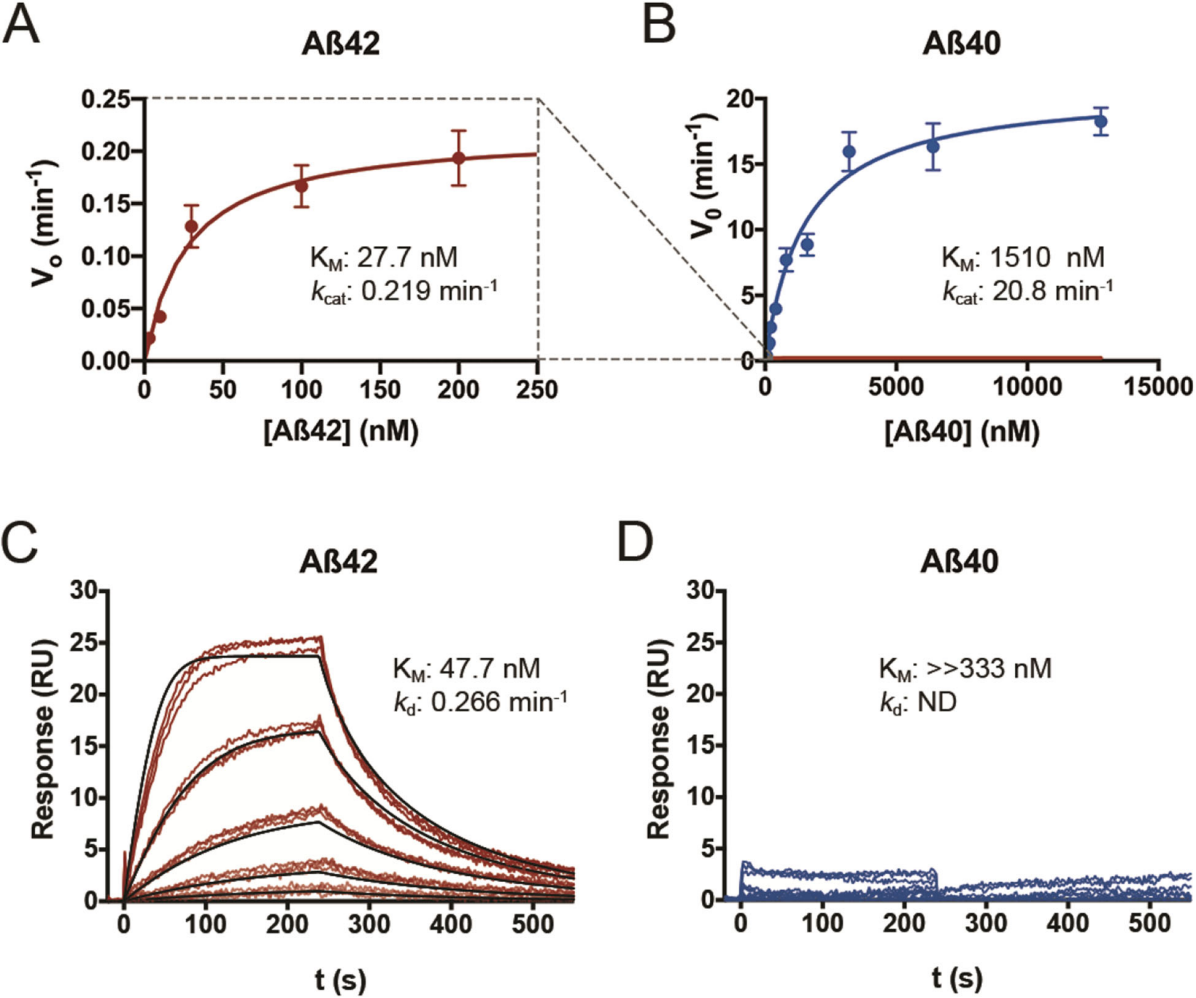
CatD degrades Aβ42 and Aβ40 with markedly different kinetics. **a**, **b** Plots of initial velocity (*v*_o_) vs. substrate concentration for Aβ42 (**a**, red) and Aβ40 (**b**, blue). The dashed lines (gray) show the relative position of the data in **a** when superimposed on the same scale as is used for the data in **b**. Quantitative kinetic parameters are provided in Supp. Table S2. **c**, **d** Surface plasmon resonance confirms that Aβ42 exhibits markedly higher affinity for CatD than Aβ40, independent of degradation. Traces obtained for 3-fold dilutions of Aβ42 (**c**) and Aβ40 (**d**) beginning at 333 nM. Analysis of the fitted curves in (**c**) yielded a *K*_M_ of 47.7 + 0.041 nM and a *k*_d_ value of 0.266 + 7.2 × 10^5^ min^−1^ for Aβ42. Consistent with the kinetics of Aβ40 binding obtained by other methods (Supp. Table S2), no significant binding of Aβ40 was observed within the conditions used. Note that, for technical reasons, these experiments were conducted at pH 4.5, precluding direct quantitative comparisons to kinetic parameters determined by other methods at pH 4.0

As a logical step in the characterization of CatD as a novel AβDP, we sought to quantify the kinetics of its degradation of Aβ42 and Aβ40 at pH 4.0. For these and all other enzymological experiments, we were careful to use freshly prepared, well-characterized batches of monomeric human Aβ42 or Aβ40 peptides, which we routinely prepared by size-exclusion chromatography [39, 40]. As assessed by multiple quantitative methods, the kinetics of Aβ42 and Aβ40 degradation were found to be strikingly dissimilar. For example, by ELISA, Aβ42 exhibited an unexpectedly strong, low-nanomolar affinity for CatD (*K*_M_ = 27.7 ± 6.0 nM), in marked contrast to Aβ40, which showed a low-micromolar value (*K*_M_ = 1.51 ± 0.26 μM) that is more typical of the interaction between Aβ and other AβDPs (Fig. 3a, b; Supp. Table S2). The *k*_cat_ values obtained for Aβ42 and Aβ40 were likewise dramatically different (0.23 ± 0.01 vs. 20.8 ± 1.1 min^−1^, respectively; Fig. 3a, b; Supp. Table S2). The *k*_cat_ value for Aβ42 (0.23 min^−1^) in particular stands out as being exceptionally low—indicating that it takes each molecule of CatD a remarkable ~ 4.3 min to process just 1 molecule of Aβ42. The results obtained by ELISA were subsequently confirmed by multiple independent enzymological methods, including trichloroacetic acid-mediated precipitation of ^125^I-labeled Aβ peptides, competition experiments with fluorogenic peptide substrates [41], and a novel homogeneous time-resolved fluorescence (HTRF)-based approach using end-specific antibodies (see *Supplemental Methods*). All of these methods yielded quantitative data in good agreement with the ELISA results (Supp. Table S2). Finally, in an independent approach, we used surface plasmon resonance to quantify the affinity and dissociation constant of Aβ42 and Aβ40 to immobilized CatD, in this case at pH 4.5. In excellent agreement with the enzymological findings, Aβ42 showed a *K*_M_ of 47.7 + 0.041 nM and a *k*_d_ value (dissociation constant, comparable to *k*_cat_) of 0.266 + 7.2 × 10^−5^ min^−1^ at pH 4.5, whereas, consistent with the other findings, the *K*_M_ for Aβ40 was outside the range of concentrations tested (> 333 nM; Fig. 3c, d).

To complete the characterization of CatD as a novel AβDP, we also investigated whether the protease was capable of degrading Aβ in various states of aggregation. On short time scales (≤ 1 days), no effect was observed on the degradation of fibrils, protofibrils, or SDS-induced oligomers of Aβ42 (Supp. Fig. S8A-C). However, over longer time scales (≥ ~ 4 days), fibrils (Supp. Fig. S8A) and protofibrils (Supp. Fig. S8B) of Aβ42 were effectively degraded by CatD at pH 4.0, but not by trypsin at pH 4.0 or IDE at pH 7.4.

### Low *K*_M_ and *k*_cat_ values render Aβ42, a potent competitive inhibitor of CatD

**Fig. 4:**
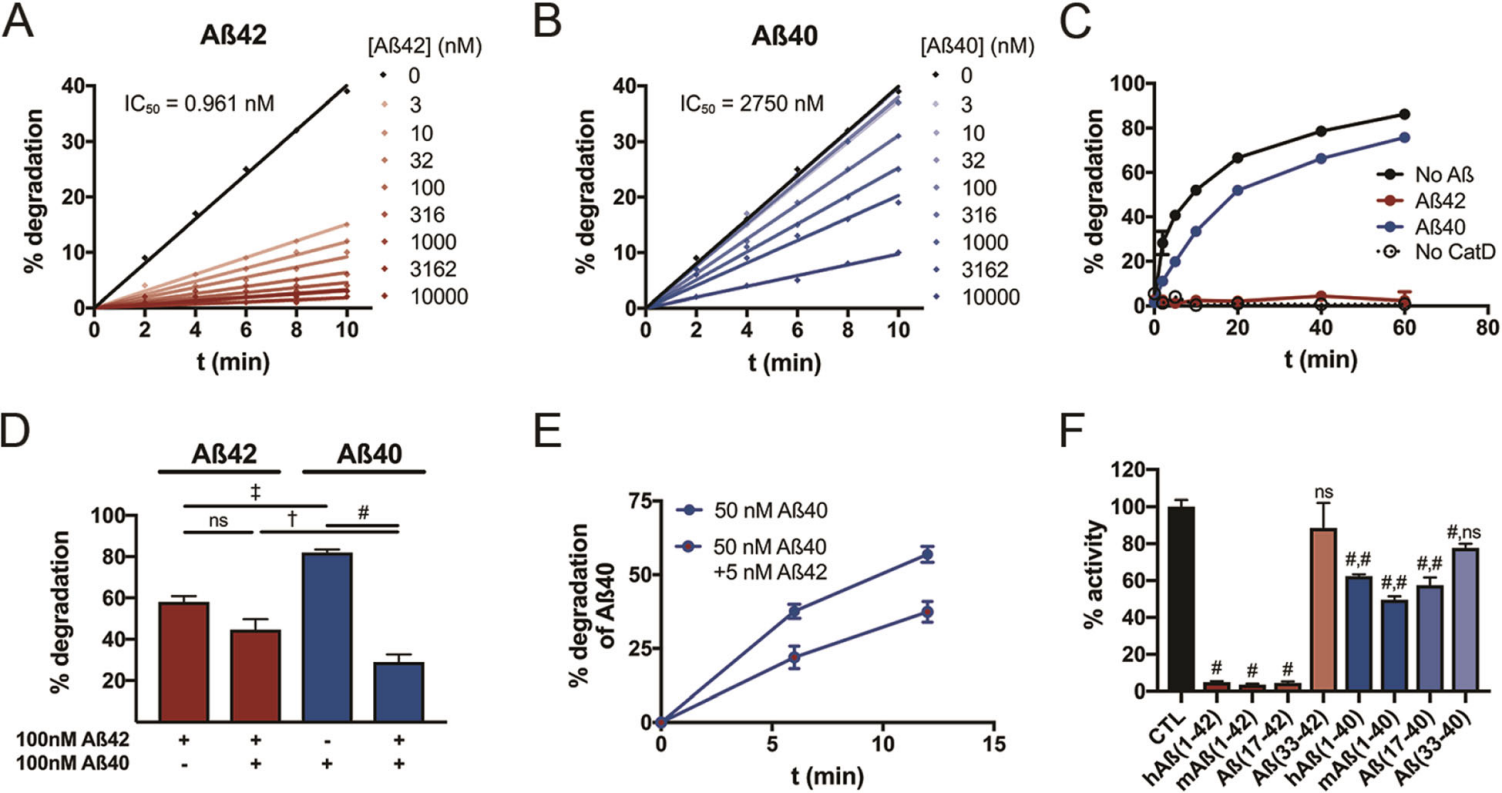
Aβ42 is a potent competitive inhibitor of CatD. **a**, **b** Competitive inhibition by Aβ42 (**a**) and Aβ40 (**b**) of CatD activity quantified by a fluorogenic substrate. Note that just 3 nM Aβ42 inhibits CatD (nominal concentration, ~ 1 nM) by more than 50%. **c** Comparable data for CatD activity quantified using an Aβ degradation assay, with 200 nM fluorescent Aβ alone (no Aβ) or in combination with 200 nM Aβ42 (red) or Aβ40 (blue). **d** Quantification of Aβ42 (red) and Aβ40 (blue) degradation either alone (100 nM) or in combination (100 nM each). Note that Aβ42 significantly inhibits Aβ40 degradation, but the converse is not true. Data are mean ± SEM for 4–8 replicates per group. ^†^*p* < 0.01; ^‡^*p* < 0.001; ^#^*p* < 0.0001. **e** Aβ40 degradation is significantly inhibited by 1/10 the concentration of Aβ42, a ratio representative of that present in vivo. **f** CatD is strongly inhibited by multiple Aβ peptides and fragments ending at position 42, including full-length murine Aβ (mAβ_(1–42)_) and the p3 fragment (Aβ_(17–42)_), more strongly than the corresponding peptides ending in Aβ40. The C-terminal fragment of Aβ42, Aβ_(33–42)_, failed to inhibit significantly, while the corresponding fragment, Aβ_(33–40)_, showed a modest but statistically significant inhibition under the conditions tested. Data are mean ± SEM for 4–8 replicates per group. ^#^*p* < 0.0001. For data on Aβ peptides ending at position 40, the 2 symbols reflect the statistical significance of comparisons to buffer-only control (CTL) and to the corresponding fragments ending at position 42, respectively

The very strong affinity (*K*_M_) of Aβ42 for CatD, combined with its exceptionally slow turnover rate (*k*_cat_), effectively renders Aβ42 a highly potent competitive inhibitor of CatD. To explore these inhibitory properties more quantitatively, we measured CatD activity in real time using a fluorescence dequenching assay in the presence of varying quantities of Aβ42 or Aβ40. Using this paradigm, Aβ40 inhibited CatD with an IC_50_ of 2.75 μM, whereas, in marked contrast, Aβ42 inhibited CatD > 10^3^ more potently, with a calculated IC_50_ of 0.96 nM (Fig. 4a, b). Given that the nominal concentration of CatD in these experiments was ~ 1 nM, this implies an essentially 1:1 interaction between Aβ42 and CatD that nevertheless potently inhibits the protease for prolonged periods. Similar results were obtained using a fluorescence polarization-based Aβ degradation assay [42], where 200 nM Aβ42 was found to essentially completely inhibit the degradation of fluorescent Aβ (200 nM) by CatD, while 200 nM Aβ40 inhibited its degradation only partially (Fig. 4c). Likewise, when Aβ42 and Aβ40 were combined together in equimolar quantities (100 nM), the degradation of Aβ42 was not slowed relative to Aβ42 alone, whereas the degradation of Aβ40 was significantly slowed relative to Aβ40 alone (Fig. 4d). In the latter experiment, we also note that Aβ42 alone was degraded more quickly than Aβ40 alone. Together, these results imply that, for a mixture of both peptides, Aβ42 is degraded more efficiently by CatD than Aβ40, providing a plausible mechanism explaining how the deletion of CatD increases the Aβ42/40 ratio. Given that Aβ42 is usually present at concentrations ~ 10-fold lower than Aβ40 in vivo, we also tested whether Aβ40 degradation could be inhibited by 1/10 as much Aβ42. In fact, the degradation of 50 nM Aβ40 was significantly inhibited by just 5 nM Aβ42 (Fig. 4e).

In a final set of experiments, we aimed to test whether the marked differences in the kinetics of human Aβ42 vs. Aβ40 degradation might extend to full-length rodent Aβ or shorter Aβ fragments ending at positions 42 vs. 40. To address this, we tested the extent to which different peptides at identical concentrations inhibited CatD activity monitored with a fluorogenic substrate. CatD activity was inhibited > 90% by 1 μM of human Aβ42, rodent Aβ42, and the α-secretase-derived p3 fragment of APP ending at position 42 (Aβ_(17–42)_), but not by a short C-terminal peptide (Aβ_(33–42)_; Fig. 4f). In contrast, the corresponding Aβ peptides ending at position 40 instead of 42 inhibited the degradation of the fluorogenic substrate to a significantly lesser extent (Fig. 4f). The result for the p3 fragment ending at position 42 is especially notable, since it is a naturally occurring product of endogenous APP processing — moreover, one that is produced at levels ~ 10-fold higher than Aβ42 [43].

## Discussion

Taken together, our findings support the twin conclusions that CatD is a key regulator of brain Aβ proteostasis in vivo and that a significant portion of Aβ is trafficked to lysosomes. CatD accounts for the vast majority of Aβ-degrading activity in soluble brain extracts; deletion of CatD in vivo results in marked increases in cerebral Aβ; and Aβ accumulates to high levels in lysosomes when CatD is absent. Collectively, these observations suggest that a significant fraction of Aβ is normally trafficked to lysosomes, where it is degraded primarily by CatD. In addition, our findings raise the compelling possibility that Aβ42/40 ratios can be regulated not only at the site of Aβ production, via presenilin/γ-secretase [44], but also via differential degradation of different length Aβ species by CatD, and perhaps also by other AβDPs.

Our results suggest that CatD may be, by several measures, the most pathologically significant AβDP yet identified. Quantitatively, the increases in endogenous Aβ42 and Aβ40 levels induced by deletion of CatD exceed those induced by deletion of any other AβDP studied to date [17, 45] or, indeed, by simultaneous deletion of multiple AβDPs [46] (see also Fig. 2d, e). Qualitatively, moreover, CatD is the only AβDP that, when deleted, has been shown to trigger the frank deposition of endogenous murine Aβ by just 3 weeks of age. These findings strongly suggest that CatD’s contribution to the overall economy of cerebral Aβ exceeds that of any previously characterized AβDP.

The involvement of CatD in the intralysosomal clearance of Aβ has potentially significant pathological implications. In particular, intracellular pools of Aβ have been hypothesized to play a disproportionately important role in AD pathogenesis [47], for example, initiating neuronal cell death at concentrations several orders of magnitude lower than extracellular Aβ [48]. Nevertheless, this has been a technically challenging field of inquiry; manipulation of CatD could provide an elegant means to assess the role of intralysosomal Aβ in the pathogenesis of AD. It is relevant to note in this context that Cheng and colleagues recently reported that the deletion of one allele of *CTSD* in APP/PS1 transgenic mice had no effect on extracellular Aβ deposits [49]. This lack of effect could have multiple potential explanations. First, it might reflect the fact that CatD only regulates intracellular pools of Aβ. Second, as our data suggest, it might instead be attributable to the apparent compensatory increases in CatD protein and activity we observed in the heterozygous state—although the decrease in CatD levels in CatD-HET mice was determined to be somewhat greater (~ 38%) in the study by Cheng and colleagues than what we found (~ 25%) [49]. Third, CatD might not be rate-limiting in the determination of cerebral Aβ levels, such that a gene dosage dependency would not be observed. Finally, we cannot entirely exclude the possibility that some other non-specific consequences of CatD deletion, perhaps involving neuronal ceroid lipofuscinosis or some other indirect consequences, could account for the increase in Aβ levels and Aβ42/40 ratios in CatD-KO mice. Given the lack of clarity on this and many other significant questions about the potential role of CatD in the pathogenesis of AD, research in this area would be greatly facilitated by future work with animal models that permit the manipulation of CatD conditionally, reversibly, and/or cell type specifically [27].

The finding that insoluble forms of Aβ were increased in CatD-KO mice while soluble forms were decreased also deserves discussion. Insoluble forms of Aβ are generally considered to represent aggregated species [31]. Notably, the aggregation of Aβ—and Aβ42 in particular—is dramatically accelerated under the acidic conditions present in the lysosomes [50]. This fact, together with our immunohistochemical findings, strongly suggests that the insoluble pool of Aβ represents aggregates of Aβ within lysosomes. As to why soluble forms of Aβ decrease in CatD-KO mice, we can only speculate, but we note that it has been shown that the presence of aggregated forms of Aβ acts to seed the aggregation of soluble pools of Aβ, thus reducing the concentration of monomeric Aβ species [51]. In this connection, it is interesting to note that NEP-KO mice showed increases in soluble Aβ, while IDE-KO mice did not, perhaps reflecting the fact that NEP is present and active within the endolysosomal system, while IDE is not [52].

The most pathologically significant, and initially the most puzzling, consequence of CatD deletion was the highly consistent increase in the cerebral Aβ42/40 ratio. Although any number of indirect mechanisms might in principle have accounted for this effect in vivo, we discovered that CatD degrades Aβ42 and Aβ40 in vitro with strikingly different kinetics, implying that these enzymological parameters could potentially be operative in vivo. Depending on the specific methodology used, the *K*_M_ of Aβ42 for CatD at pH 4.0 was estimated to be from 3.2 to 28 nM, or from ~ 50 to ~ 600 times stronger than that for Aβ40 (Supp. Table S2). The turnover number (*k*_cat_) of Aβ42 was found to be unexpectedly slow, as well, with different methodologies yielding estimates of 0.22 to 1.1 min^−1^ (Supp. Table S2). These values are from ~ 40- to ~ 110-fold lower than the corresponding values for Aβ40 and, quite significantly, are 10^2^- to 10^3^-fold slower than the *k*_cat_ of Aβ40 degradation by IDE, neprilysin, and plasmin (calculated from [42]). Expressed differently, the processing of one molecule of Aβ42 requires the same amount of time as the processing of 10^2^ to 10^3^ molecules of Aβ40 by CatD or other well-characterized proteases.

Taken together with the strong affinity of Aβ42 for CatD, the slow turnover number essentially renders Aβ42 a very potent inhibitor of CatD, as confirmed by multiple experiments in this study. The possibility that aggregation of Aβ42 accounts for its potent inhibitory power is excluded by several observations. First, we showed that a mere 3 nM of monomeric Aβ42 inhibits 1 nM of CatD by > 50%. If Aβ42 were in the form of aggregates, their average molarity would be decreased relative to the monomeric state, making such a potent interaction physically impossible. Second, in the ELISA-based degradation experiments, we obtained absolute concentrations of Aβ in agreement with the nominal monomeric Aβ concentrations. Third, both murine Aβ42 and p3 fragments ending at position 42—which are both far less prone to aggregation than full-length human Aβ—were also shown to be effective inhibitors of CatD. Finally, the possibility that Aβ aggregated significantly when exposed to pH is similarly ruled out. Aggregation, if it did occur during the course of the degradation reactions, would *decrease* the apparent concentration of Aβ detected by ELISA, thereby resulting in an overestimate of the rates of degradation; to the contrary, Aβ42 levels remained quite stable throughout the course of the reactions, particularly for the highest concentrations. Collectively, these observations strongly suggest that Aβ42 potently inhibits CatD in an aggregation-independent manner.

Our findings imply an intriguing bidirectional relationship between Aβ42 and CatD activity. On the one hand, impaired CatD activity can trigger selective increases in Aβ42, and on the other hand, Aβ42—and the corresponding p3 fragment—can competitively inhibit CatD activity, in some instances with exquisite potency. This bidirectional interrelationship is especially notable from a pathological perspective and gives rise to some novel—albeit speculative—possibilities. Given that defects in CatD can trigger multiple neurodegenerative diseases [10], it is reasonable to ask whether the central role of elevated Aβ42 in AD pathogenesis may, in part, involve its potent ability to competitively inhibit CatD. While speculative, such a mechanism could conceivably be operative in the poorly understood link between elevated Aβ42 concentrations and tauopathy. In this context, it is especially notable that tau is degraded by CatD in vitro [9], and there is accruing evidence that disruptions to lysosomal clearance of tau may play a role in tau accumulation [5]. Moreover, the deletion of CatD in *drosophila* was shown to exacerbate the premature lethality induced by neuronal overexpression of tau [53], suggesting that CatD may also protect against the pathological effects of tau. These findings, together with those of the present study, strongly suggest that CatD normally plays a protective role in AD, a function that can be selectively compromised by elevated concentrations of Aβ42.

There are many limitations inherent in the use of CatD-KO mice, due to their premature lethality and their development of profound neurodegeneration and lipofuscinosis. A proper assessment of the role of CatD in the pathogenesis of AD will require more sophisticated means for manipulating CatD. Because aging is the primary risk factor for AD—and because recent findings show that the maturation of and post-translational modifications of CatD can change in an age-dependent manner [54]—inducible expression systems will likely be needed.

If, as we propose, CatD plays a protective role in AD by virtue of a functional role as an AβDP, then we would predict that loss-of-function mutations in CatD would increase the risk for AD. In fact, a large number of genetic association studies have investigated a single-nucleotide polymorphism present in exon 2 of the *CTSD* gene (rs17571; C➔T224), which leads to an Ala➔Val transition within the prodomain of the CatD zymogen (Supp. Fig. S9A), and which has been reported to perturb the maturation and trafficking of CatD [55]. Considered individually, these studies have yielded conflicting results. However, using data from AlzGene [56], a meta-analysis of all 18 Caucasian-only reports published to date, excluding those with Hardy-Weinberg equilibrium violations, yields a statistically significant odds ratio estimate for the rs17571 polymorphism (OR = 1.20, 95% CI = 1.01–1.42, *p* = 0.038) (Supp. Fig. S9B). Although the effect size of this association is comparatively modest, it is critical to emphasize that the functional consequences of this mutation are predicted to be relatively subtle, given that the rs17571 polymorphism results in a conservative amino acid substitution (A58V) in a non-functional, poorly conserved region of the latent CatD zymogen (Supp. Fig. S9A). The finding that such a subtle mutation nevertheless confers a statistically significant increase in AD risk lends support to the idea that CatD may play a relatively important pathophysiological role in the etiology of AD, as would be predicted from the functional findings of the present study.

## Conclusion

In conclusion, the totality of our results supports the hypothesis that CatD plays a protective role in the pathogenesis of AD by regulating intralysosomal Aβ levels as well as Aβ42/40 ratios through differential degradation of Aβ42 and Aβ40, an effect that is driven by aggregation-independent, enzymological mechanisms. More speculatively, the finding that Aβ42 competitively inhibits CatD at pathophysiologically relevant concentrations suggests a possible molecular mechanism linking elevations in Aβ42 to downstream neuropathological sequelae characteristic of AD.

## Methods/experimental

### Aim, design, and setting

The objective of the present study was to evaluate the role of CatD in Aβ proteostasis in vivo and to more completely characterize its Aβ-degrading function. To that end, homogenized brain extracts from 15- to 26-day-old CatD-KO, CatD-HET, and CatD-WT mice were analyzed for Aβ-degrading activity, protein levels, and steady-state soluble and insoluble Aβ levels. Paraffin-embedded brain tissue from these mice was analyzed by immunohistochemistry for AD-related markers. Cultured embryonic (E18) hippocampal neurons were analyzed for Aβ secretion into the conditioned medium and the uptake and catabolism of fluorescently tagged synthetic Aβ peptides. Mass spectrometry was conducted to analyze the fragments of synthetic Aβ peptide fragments generated by recombinant CatD. Degradation of aggregated Aβ42 by recombinant CatD was assessed by thioflavin T fluorescence and western blotting. A variety of proteolytic degradation assays were performed in the absence or presence of different Aβ and p3 fragments, and binding assays were performed by surface plasmon resonance, all with recombinant human CatD. The research was conducted in multiple state-of-the-art biomedical laboratories.

### Animals

Mice were bred and housed in AAALAC-accredited facilities in accordance with the National Institutes of Health Guidelines for the Care and Use of Laboratory Animals. CatD-KO [26], IDE-KO [21], NEP-KO [19], APP-KO [57], BACE1-KO [58], and Twitcher mice [34] were maintained as inbred lines, each in a mixed C57Bl/6J, DBA genetic background. The NEP/IDE-DKO line was derived from crosses between the NEP-KO and IDE-KO lines. Analyses were restricted to age- and sex-matched groups of littermates for all genotypes, except NEP/IDE-DKO mice, which were compared to age-and sex-matched NEP-WT and IDE-WT mice grouped together for statistical analysis. Due to the premature lethality present in CatD-KO mice, we focused our analyses on tissues extracted from 15- to 26-day-old mice, using age- and sex-matched littermate WT controls in all cases except the NEP/IDE-DKO line, for which littermate controls WT at both loci could not be obtained. These mice were instead compared to a group of both NEP-WT and IDE-WT animals, which did not differ significantly from one another in terms of any analyte examined.

### Aβ quantification

Endogenous murine Aβ40 and Aβ42 were extracted from frozen hemibrains with 0.2% diethylamine (DEA) and guanadinium isothiocyanate, as described [59], then quantified using Aβ42 and Aβ40 end-specific sandwich ELISAs (Wako) [31]. For Aβ quantification in neuronal media, conditioned medium was supplemented with Complete Protease Inhibitor Cocktail (Roche) and analyzed without further extraction using in-house ELISA systems based on antibody pairs 33.1.1/13.1.1 and 2.1.3.35.86/33.1.1, respectively [30, 31]. All ELISA measurements of brain Aβ were normalized to the average background signal obtained from APP-KO and BACE1-KO mouse brains processed and analyzed in parallel with other samples.

### Enzymological studies

For the determination of the pH dependence of Aβ degradation in soluble brain extracts, freshly harvested brain tissue from 15-day-old mice was dissociated in 20 mM Tris-HCl, pH 7.4 at 4 °C using a Dounce homogenizer, then centrifuged at 1000×*g*. The resulting supernatant was diluted 1:20 in Britton-Robinson buffers of different pHs, and CatD activity was quantified either using a well-characterized fluorescence polarization-based Aβ degradation assay as described [42] or by monitoring hydrolysis of the CatD-specific fluorogenic substrate, Mca-GKPILFFRLK-Dnp. Kinetic experiments were conducted using freshly prepared, monomeric Aβ peptides separated from aggregated species by size-exclusion chromatography (SEC) and characterized as described [39, 40]. Aβ peptides and PepA were diluted in neutral Dilution Buffer (20 mM Tris, pH 8.0 supplemented with 0.1% BSA), with addition of DMSO as appropriate, and reactions were initiated by transfer into Assay Buffer (60 mM Na-citrate; 80 mM Na_2_HPO_4_, pH 4.0; Sigma) supplemented with purified human CatD (Enzo Life Sciences). Where required, reactions were terminated by adjustment to neutral pH with 10× Stop Buffer (0.2 M Tris-HCL, pH 9.5 supplemented with 10 μM PepA). For ELISA-based experiments, Aβ42 and Aβ40 were quantified by well-characterized sandwich ELISAs (Wako) [29]. Competitive inhibition experiments were conducted using either ELISAs, an Aβ-degradation assay [42] or the fluorogenic substrate.

### Surface plasmon resonance

Binding studies were performed using a Biacore S51 optical biosensor equipped with a CM5 sensor chip. Purified human CatD (Enzo Life Sciences) was diluted to 0.1 nM in Coupling Buffer (10 mM NaAc, pH 4.25) and amine-coupled to the chip surface. Aβ peptides were diluted in Running Buffer (50 mM Na-Citrate, 200 mM NaCl, 1 mM EDTA, 2 mM DTT, 0.005% Tween-20, pH 4.5) and tested in triplicate using a 3-fold dilution series beginning at 333 nM. Binding data were fitted to a simple 1:1 interaction model using manufacturer-supplied software (Biacore). Kinetic parameters were obtained by analysis of fitted curves using Anabel [60].

### Statistical analyses

Tests of significance between individual experimental and control groups were conducted using unpaired *t* tests, after *F* tests for equality of variances. For data in two or more groups and/or also containing another variable (e.g., age), mixed-effects analysis via ANOVA was performed. Group sizes were determined by power analysis of comparable historical experimental data sets, using the Student’s *t* test with the alpha level set at 0.05. All calculations were performed from the raw data in Prism 8 for Mac OS (Graphpad Software, LLC).

## Supplementary information

**Additional file 1:Table S1.** Determination of CatD-mediated cleavage sites within Aβ42 and Aβ40 via mass spectrometry. **Table S2.** Kinetics of Aβ42 vs Aβ40 degradation at pH 4.0 quantified by several independent methods. **Fig. S1.** The mechanism by which CatD regulates Aβ levels does not involve effects on APP, Aβ production or known Aβ-degrading proteases. **Fig. S2.** Soluble Aβ42 and Aβ40 levels in CatD-KO, −HET and -WT brains. **Fig. S3.** Cerebral Aβ levels are unchanged in another mouse model featuring profound lysosomal dysfunction and premature lethality. **Fig. S4.** Immunohistochemical analysis of CatD-KO mice shows selective accumulation of Aβ42 in lysosomes and other intracellular compartments by 3 weeks of age. **Fig. S5.** Studies in primary embryonic cultured neurons. **Fig. S6.** Mass spectra of Aβ42 degradation by CatD. **Fig. S7.** Mass spectra of Aβ40 degradation by CatD. **Fig. S8.** Activity of CatD against aggregated Aβ species. **Fig. S9.** Evidence for a statistically significant genetic association between a functional polymorphism in *CTSD* and risk for late-onset AD (LOAD).

## Data Availability

All data generated or analyzed during this study are included in this published article and its supplementary information files.

## Abbreviations

AD: Alzheimer disease
Aβ: Amyloid β-protein
AβDP: Aβ-degrading protease
APP: Amyloid precursor protein
CatD: Cathepsin D
*CTSD*: Cathepsin D gene
*GALC*: Galactosylceramidase gene
HET: Heterozygous
IDE: Insulin-degrading enzyme
*k*_cat_: Turnover number
*K*_M_: Michaelis-Menten constant
KO: Knockout
NEP: Neprilysin
PepA: Pepstatin A
p3: Alpha/gamma-secretase-derived fragment of APP
WT: Wild-type
