# Supplementary material for: Cathepsin D regulates cerebral Aβ42/40 ratios via differential degradation of Aβ42 and Aβ40
Source: Alzheimers Res Ther. 2020 Jul 6;12:80. doi: 10.1186/s13195-020-00649-8 (PMC7339583; doi:10.1186/s13195-020-00649-8)
Supplement: Supplementary file 1 — Additional file 1:Table S1. Determination of CatD-mediated cleavage sites within Aβ42 and Aβ40 via mass spectrometry. Table S2. Kinetics of Aβ42 vs Aβ40 degradation at pH 4.0 quantified by several independent methods. Fig. S1. The mechanism by which CatD regulates Aβ levels does not involve effects on APP, Aβ production or known Aβ-degrading proteases. Fig. S2. Soluble Aβ42 and Aβ40 levels in CatD-KO, −HET and -WT brains. Fig. S3. Cerebral Aβ levels are unchanged in another mouse model featuring profound lysosomal dysfunction and premature lethality. Fig. S4. Immunohistochemical analysis of CatD-KO mice shows selective accumulation of Aβ42 in lysosomes and other intracellular compartments by 3 weeks of age. Fig. S5. Studies in primary embryonic cultured neurons. Fig. S6. Mass spectra of Aβ42 degradation by CatD. Fig. S7. Mass spectra of Aβ40 degradation by CatD. Fig. S8. Activity of CatD against aggregated Aβ species. Fig. S9. Evidence for a statistically significant genetic association between a functional polymorphism in CTSD and risk for late-onset AD (LOAD). [file 13195_2020_649_MOESM1_ESM.docx]

### ­­Supplemental information for

**Cathepsin D regulates cerebral Aß42/40 ratios via differential degradation of Aß42 and Aß40**

Caitlin N. Suire, Samer O. Abdul-Hay, Tomoko Sahara, Dongcheul Kang, Monica K. Brizuela, Paul Saftig, Dennis W. Dickson, Terrone L. Rosenberry and Malcolm A. Leissring

**Supplemental Information Inventory**

**Table S1.** Determination of CatD-mediated cleavage sites within Aß42 and Aß40 via mass spectrometry.

**Table S2.** Kinetics of Aß42 vs Aß40 degradation at pH 4.0 quantified by several independent methods.

**Figure S1.** The mechanism by which CatD regulates Aß levels does not involve effects on APP, Aß production or known Aß-degrading proteases.

**Figure S2.** Soluble Aß42 and Aß40 levels in CatD-KO, -HET and -WT brains.

**Figure S3.** Cerebral Aß levels are unchanged in another mouse model featuring profound lysosomal dysfunction and premature lethality.

**Figure S4.** Immunohistochemical analysis of CatD-KO mice shows selective accumulation of Aß42 in lysosomes and other intracellular compartments by 3 weeks of age.

**Figure S5.** Studies in primary embryonic cultured neurons.

**Figure S6.** Mass spectra of Aß42 degradation by CatD.

**Figure S7.** Mass spectra of Aß40 degradation by CatD.

**Figure S8.** Activity of CatD against aggregated Aß species.

**Figure S9.** Evidence for a statistically significant genetic association between a functional polymorphism in *CTSD* and risk for late-onset AD (LOAD).**Table S1.** Determination of CatD-mediated cleavage sites within Aß42 and Aß40 via mass spectrometry.

**
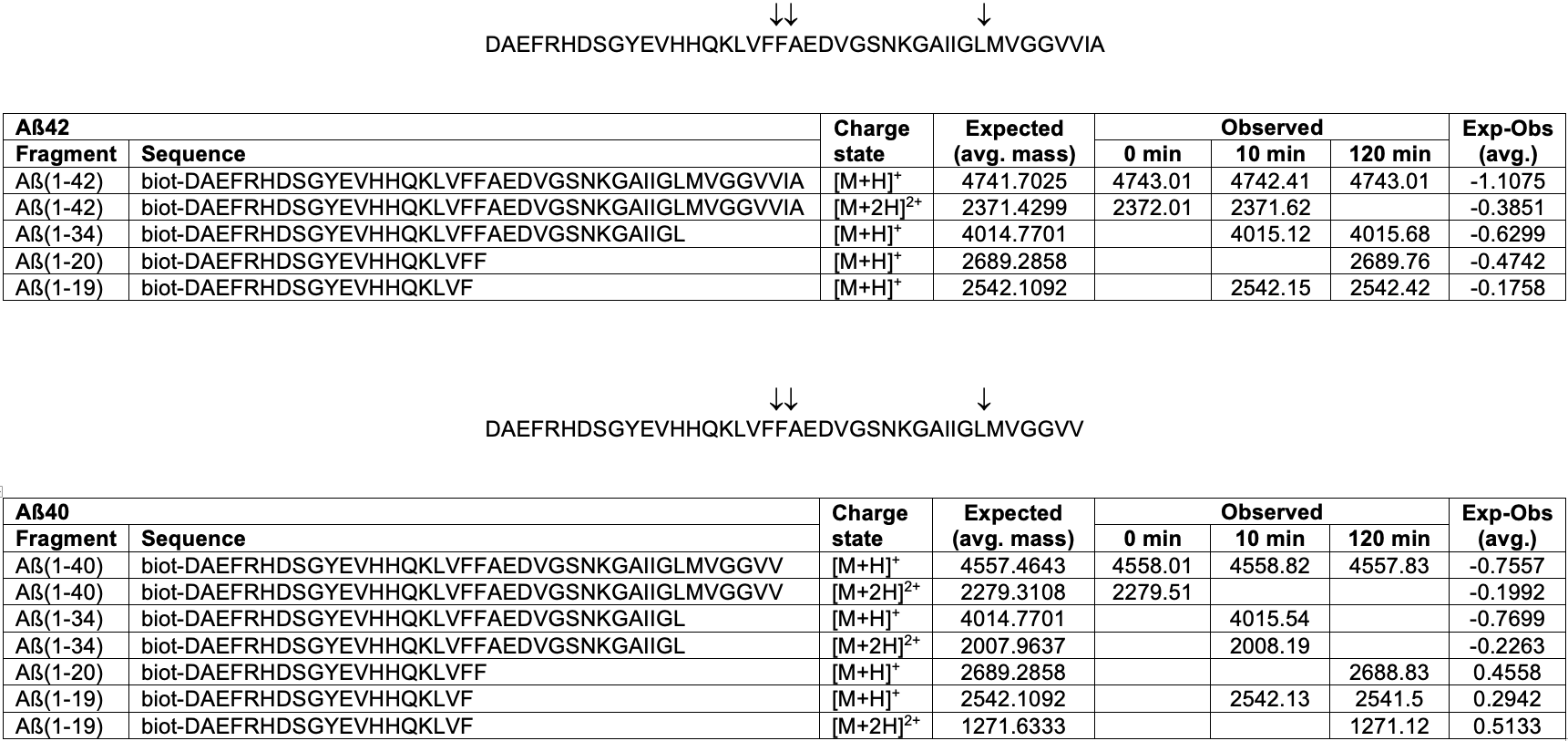
**

**Table S2. Kinetics of Aß42 vs. Aß40 degradation by CatD at pH 4.0 quantified by several independent methods.**

| **Aß42 catabolism** | **ELISA** | **^125^I-Aß42** | **silent substrate** | **HTRF** |
| --- | --- | --- | --- | --- |
| **K_M_ (nM)** | 27.7 ± 6.0 | 7.34 ± 1.64 | 3.17 ± 0.62 | 24.1 ± 4.6 |
| ***k*_cat_ (min^-1^)** | 0.219 ± 0.011 | 0.401 ± 0.087 | 0.55 ± 0.097 | 1.13 ± 0.10 |
| ***k*_cat_/K_M_ (M^-1^ min^-1^)** | 1.38 x 10^7^ | 1.24 x 10^7^ | 3.18 x 10^7^ | 6.34 x 10^7^ |

| **­Aß40**  **catabolism** | **ELISA** | **^125^I-Aß40** | **silent substrate** | **HTRF** |
| --- | --- | --- | --- | --- |
| **K_M_ (nM)** | 1510 ± 260 | 1240 ± 180 | 1880 ± 330 | 1490 ± 230 |
| ***k*_cat_ (min^-1^)** | 20.8 ± 1.1 | 15.4 ± 3.6 | 59.8 ± 7.9 | 94.8 ± 13 |
| ***k*_cat_/K_M_ (M^-1^ min^-1^)** | 0.79 x 10^7^ | 5.46 x 10^7^ | 1.74 x 10^7^ | 4.58 x 10^7^ |

**Figure S1**


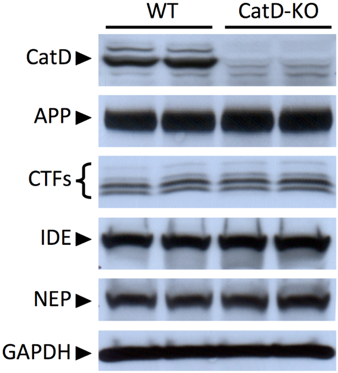


**Figure S1. The mechanism by which CatD regulates Aß levels does not involve effects on APP, Aß production or known Aß-degrading proteases.** Figure shows relative levels of CatD, the amyloid precursor protein (APP), C-terminal fragments of the latter (CTFs) that include the immediate precursors to Aß, insulin-degrading enzyme (IDE), neprilysin (NEP) and, as a loading control, GAPDH.

**Figure S2**

**

**

**Figure S2. Soluble Aß42 and Aß40 levels in CatD-KO, -HET and -WT brains.** ***A***,***B***, Levels of soluble, endogenous brain Aß42 (***A***) and Aß40 (***B***) in CatD-KO, -HET and ‑WT mice as a function of age. Data are mean ± SEM for 4-6 replicates per group. ^†^*p* <0.01; ^‡^*p* <0.001. ***C***,***D***, Soluble Aß42/40 ratios (***C***) are significantly decreased in CatD-KO mice relative to WT controls, whereas total (combined soluble and insoluble Aß) Aß42/40 ratios are increased (**D**). Note that both soluble and total Aß42/40 ratios are not significantly changed in NEP-KO, IDE-KO or NEP/IDE-DKO mice relative to their line-specific WT controls. Data are mean ± SEM for 28-30 replicates per group for CatD-KO and -WT mice and 5-11 replicates per group for the other genotypes. ^#^*p* <0.0001. ***E***,***F***, Levels of soluble, endogenous brain Aß42 (***E***) and Aß40 (***F***) in 15‑ and 26-d-old CatD-KO mice as compared to 26-d-old NEP-KO IDE-KO and NEP/IDE-DKO mice and their respective controls. Data are mean ± SEM for 4-6 replicates per group. **p*<0.05; ^†^*p* <0.01; ^‡^*p* <0.001; ^#^*p* <0.0001. **Figure S3**

**
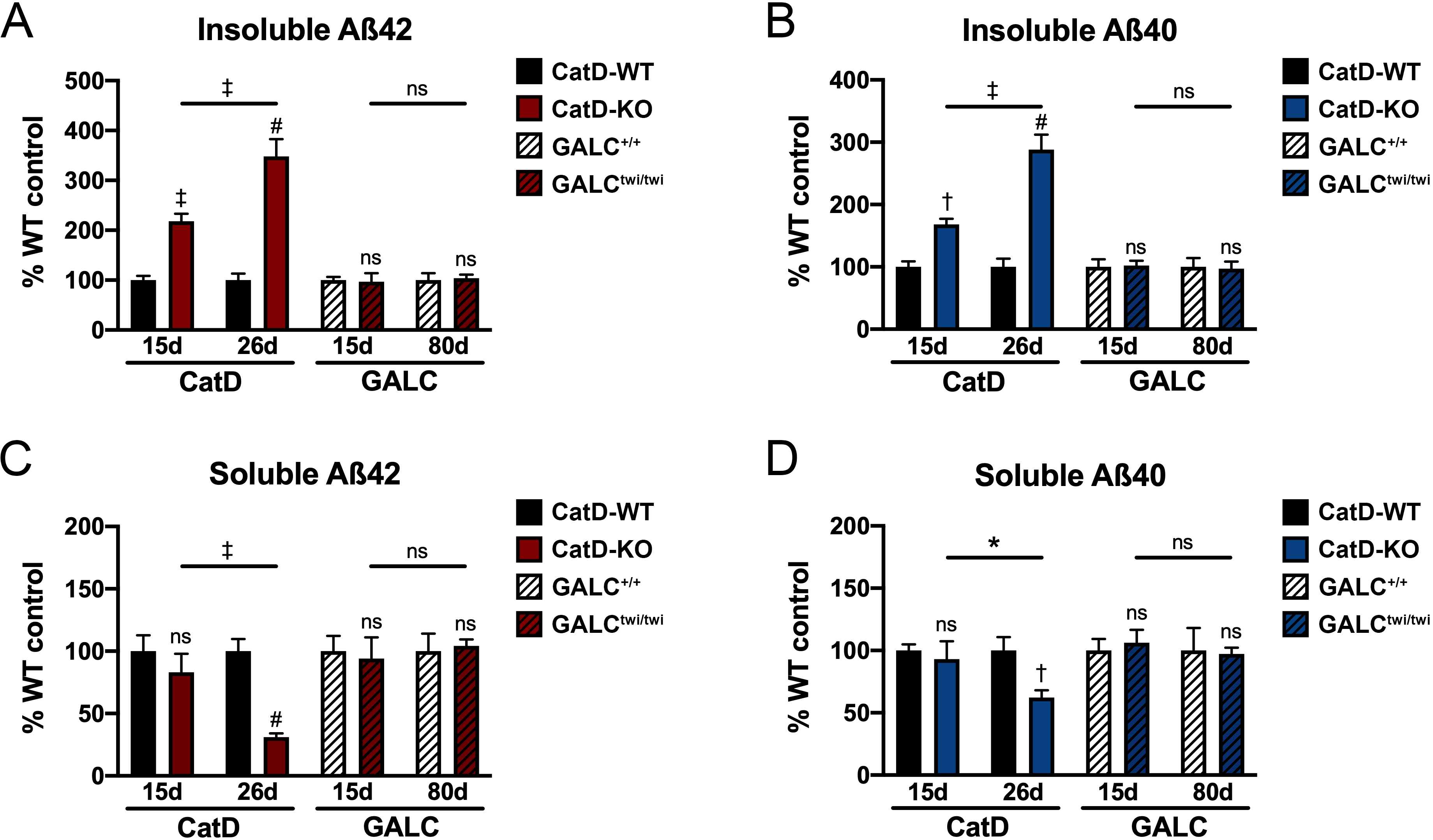
**

**Figure S3. Cerebral Aß levels are unchanged in another mouse model featuring profound lysosomal dysfunction and premature lethality.** ***A***,***D,*** Levels of endogenous insoluble Aß42 (***A***) and Aß40 (***B***) as well as soluble Aß42 (***C***) and Aß40 (***D***) in CatD-KO and GALC^twi/twi^ (*Twitcher* mouse) brains relative to strain-specific WT littermate controls at different ages. Note that GALC^twi/twi^ mice at 80 d of age are at a similar state of moribundity as CatD-KO mice at 26 d of age. Data are mean ± SEM for 6 replicates per group. **p*<0.05; ^†^*p* <0.01; ^‡^*p* <0.001; ^#^*p* <0.0001.

**Figure S4**

**
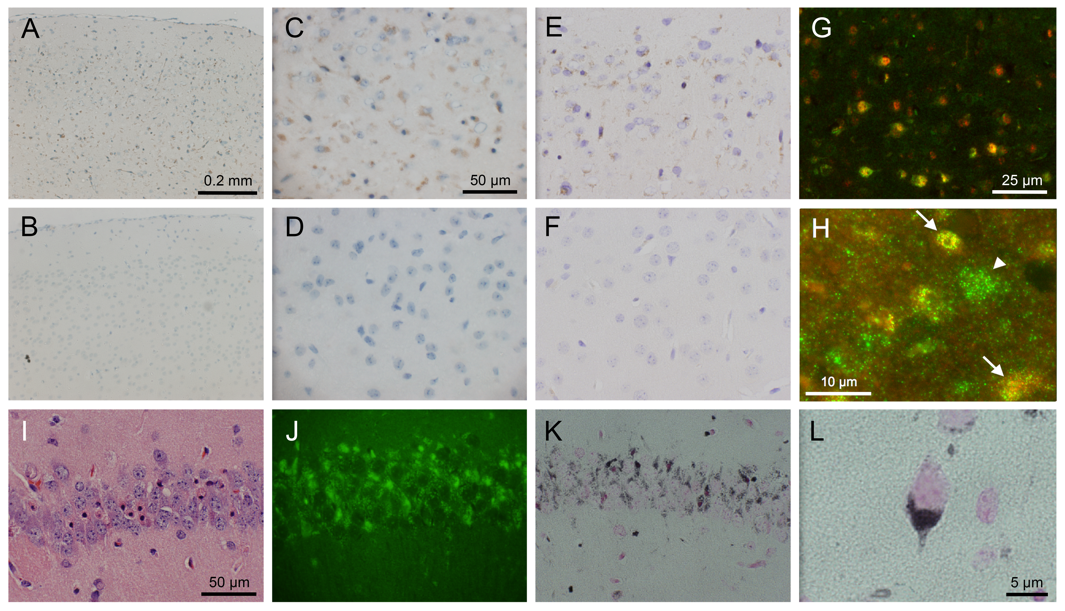
**

### Figure S4. Immunohistochemical analysis shows selective accumulation of Aß42 in lysosomes and other intracellular compartments of CatD-KO mice by 3 weeks of age. *A*-*F*, Intracellular accumulation of Aß42 detected in brains of CatD-KO (*A*, *C*, *E*) but not WT (*B*, *D*, *F*) mice using well-characterized end-specific antibodies from Wako (*A*-*D*) and Mayo Clinic (*E*,*F*). *G*,*H*, Colocalization of Aß42 (*red*) and the lysosomal marker Lamp2 (*green*) in cortical layers III – V of CatD-KO brain. *H*, Higher resolution image showing that the overlap between Aß42 and Lamp2 in some cells (*arrows*); notably, a subset of cells show no deposition of Aß42 (*arrowhead*). *I*‑*L*, Hippocampal CA1 pyramidal layer in a 3-week-old CatD-KO mouse stained with hematoxylin and eosin (*I*), thioflavin S (*J*), and Gallyas silver stain (*K*,*L*). Note the presence of pyknotic cells with condensed nuclei (*I*) and the accumulation of thioflavin- (*J*) and Gallyus- (*K*,*L*) positive staining in perinuclear regions of pyramidal neurons.

**Figure S5**


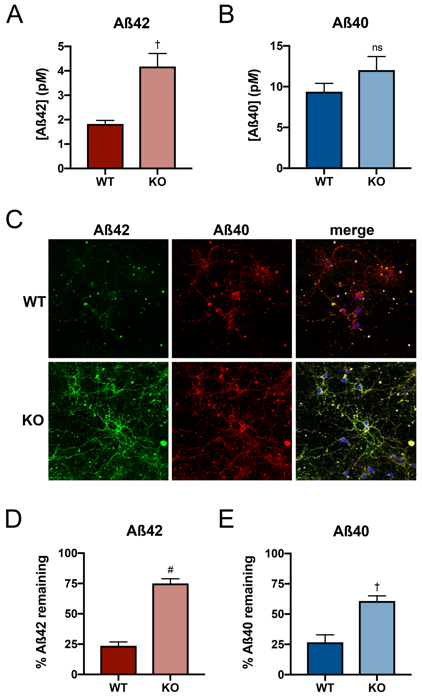


**Figure S5.** Studies in primary embryonic cultured neurons. ***A***,***B***, Aß42 (***A***) and Aß40 (***B***) levels in the conditioned medium of cultured neurons from CatD knockout (KO) mice and wildtype (WT) controls. Note that KO neurons secrete significantly more Aß42 than WT controls (***A***) and, although a trend towards higher Aß40 levels is evident, it does not achieve statistical significance. Data are mean ± SEM for 4 replicates. ^†^*p* <0.01. ***C***, Representative images of exogenously applied, fluorescently tagged Aß42 (*green*) or Aß40 (*red*) remaining in WT and KO cultured neurons after loading and subsequent 2-h incubation (see *Supplemental* *Methods* for details). ***D***,***E***, Percent Aß42 (***D***) and Aß40 (***E***) remaining after 2-h incubation, relative to freshly loaded neurons immediately after washing. Data are mean ± SEM for 6 replicates. ^#^*p* <0.0001; ^†^*p* <0.01.

**Figure S6**


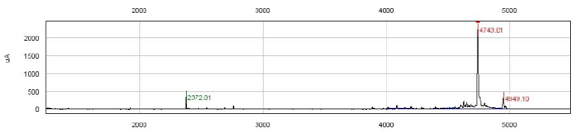
A


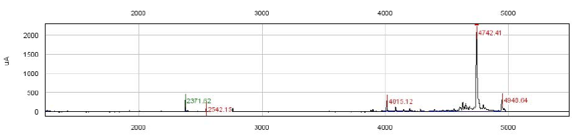
B


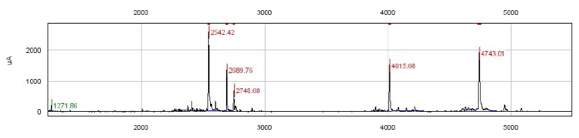
C

**Figure S6. Mass spectra of Aß42 degradation by CatD. *A****-****C***, SELDI spectra of biotinylated Aß42 at t=0 (***A***) or after incubation with recombinant human CatD for 10 min (***B***) or 2 h (***C***). Numbers in *red* and *green* reflect m/z values corresponding to charge states [M+H]^+^ and [M+2H]^2+^, respectively.

**Figure S7**


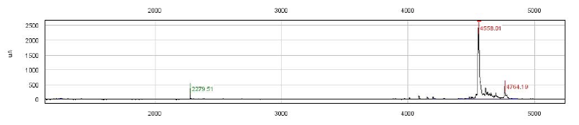
A


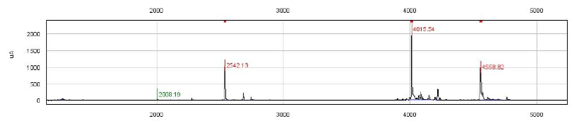
B


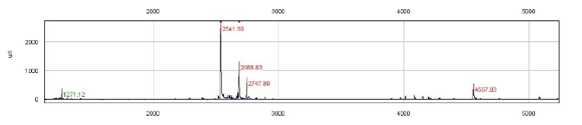
C

**Figure S7. Mass spectra of Aß40 degradation by CatD. *A****-****C***, SELDI spectra of biotinylated Aß40 at t=0 (***A***) or after incubation with recombinant human CatD for 10 min (***B***) or 2 h (***C***). Numbers in *red* and *green* reflect m/z values corresponding to charge states [M+H]^+^ and [M+2H]^2+^, respectively.

**Figure S8**

**
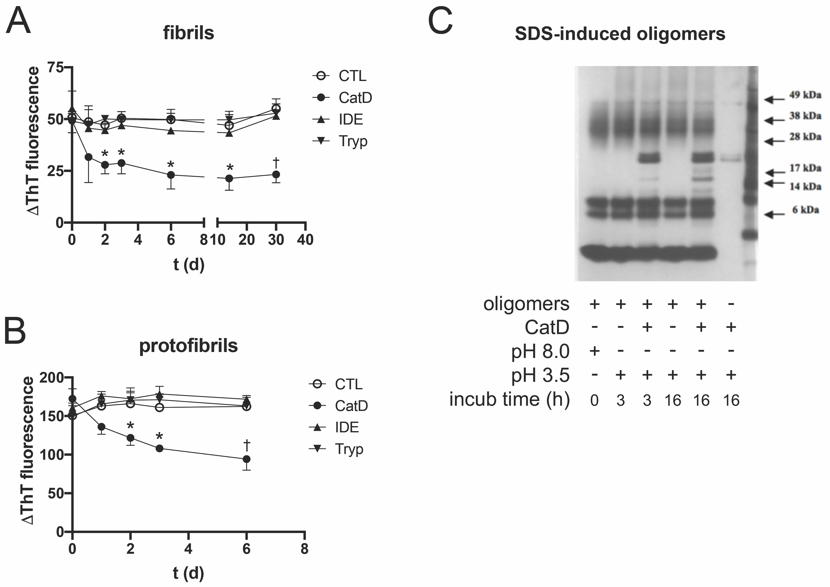
**

**Figure S8. Activity of CatD against aggregated Aß species.** ***A***,***B***, Aß fibrils (***A***) and protofibrils (***B***) are degraded by CatD at pH 4.0 as assessed by thioflavin T (ThT) fluorescence, albeit on a time-scale of days. As controls, we also tested trypsin (tryp) at pH 4.0 and IDE at pH 7.4. The protease-free control (CTL) shown is for pH 4.0; another control for pH 7.4 showed similar results, but has been omitted for clarity. See *Supplemental Methods* for details. Data are mean ± SEM for 4 independent replications. **p* <0.05; ^†^*p* <0.01. ***C***, CatD shows no effect on SDS-induced Aß oligomers, at least after 16-h incubation. See *Supplemental Methods* for details.

**Figure S9**

A


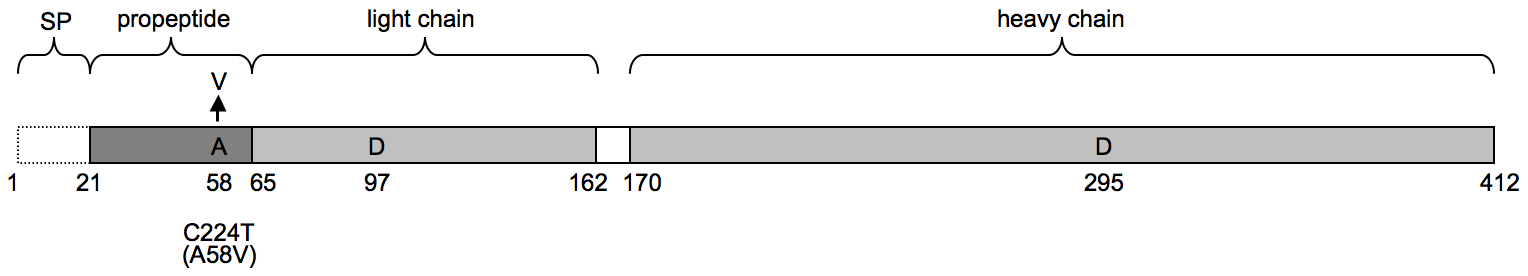


B


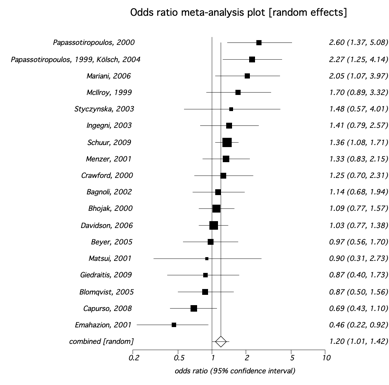


**Figure S9.** **Evidence for a statistically significant genetic association between a functional polymorphism in *CTSD* and risk for late-onset AD (LOAD).**  ***A***, Cartoon showing the location of the C224🡪T/A58V polymorphism in relation to other relevant features of the CatD molecule including the active-site aspartyl residues. SP = signal peptide. ***B***, Forest plot showing odds ratios (*black squares*), 95% confidence limits (*lines*), and the summary measure (*diamond*) for the association between C🡪224T and LOAD in 18 published Caucasian series from AlzGene [1] composed of 4,154 ADs and 9,302 controls, excluding series with Hardy-Weinberg disequilibrium in controls at p<0.01 . To perform a meta-analysis of all published, Caucasian series, allelic counts were calculated using the reported allelic frequency and sample size information from the AlzGene website [1]. Test statistics are reported for each series and pooled test statistics are reported using the random effects model (DerSimonian-Laird). Note that meta-analysis of the combined 18 series reveals a statistically significant association (*p* <0.05), with the loss-of-function SNP conferring increased risk for LOAD.

**Supplemental Methods**

**Western Blotting**

Western blots were performed as described [2], using the following antibodies: 𝛼CatD (Santa Cruz Biotechnology, Inc.), IDE-1 [3] (generous gift of D. Selkoe), C20 (reactive with the carboxy terminus of APP; generous gift of D. Selkoe), 𝛼NEP antibody 56C6 (Santa Cruz Biotechnology, Inc.), and 𝛼GAPDH (Alpha Diagnostic Intl.).

### Immunohistochemistry

Brains were collected following transcardial perfusion with PBS, fixed in 10% formalin solution, then embedded in paraffin. Parasagittal sections (5 µm) were deparaffinized in a graded alcohol series, and endogenous peroxidase activity was quenched by treatment with 3% H_2_O_2_ for 5 min prior to staining. Primary antibodies used (and their sources and dilutions) were: 2.1.3.35.86 (Mayo; 1:1000); 13.1.1 (Mayo; 1:1000); BC05 (Wako; 1:1000); BA27 (Wako; 1:1000); Lamp2 (1:2000; AbCam). For immunohistochemical staining, the primary antibodies were detected using appropriate HRP-conjugated secondary antibodies (Novus Biologicals; 1:5000). For immunofluorescence studies, endogenous fluorescence was quenched using 1% Sudan Black-B (SPI Supplies), and primary antibodies were detected with Alexa Fluor^TM^-tagged secondary antibodies (Invitrogen; 1:5000). Staining with hematoxylin/eosin, thioflavin S, and Gallyus silver stain were conducted as described [4].

### Live cell imaging and Aß quantification

Hippocampus-enriched neurons were cultured as described **[5]** from individual E18 embryos derived from a cross between CatD-HET mice, and plated (10^6^ cells/cm^2^) on 35-mm poly-D-lysine-coated glass bottom culture dishes (MatTek Corp.). After maintenance for 7 to 10 days in Neurobasal medium containing the growth supplement B27 (Invitrogen). For quantification of secreted Aß, cells were washed twice, then incubated in the latter medium overnight, and the conditioned medium was collected, supplemented with Complete Protease Inhibitor Cocktail (Roche) and analyzed without further extraction using in-house ELISA systems based on antibody pairs 33.1.1/13.1.1 and 2.1.3.35.86/33.1.1. Aß uptake and catabolism was assessed by washing neurons twice, then incubated in the growth medium containing a mixture of Aß42 and Aß40 (500 nM each) labeled N-terminally with HiLyte Fluor^TM^ 488 or HiLyte Fluor^TM^ 555, respectively (Anaspec). Following incubation at 37 ^o^C/5% CO_2_ for 2 h, cells were washed 3 times, imaged by confocal microscopy (see below), returned to 37 ^o^C/5% CO_2_ for 2 h, stained with Hoescht 33258 (Invitrogen), washed and imaged again. Images were obtained using the 488-nm and 543-nm laser lines on a Zeiss LCM 510 confocal microscope the intensities of which were calibrated and maintained constant for all imaging. A computer-operated stage (Zeiss) was used to record the position of imaged cells and to facilitate their subsequent location, which was verified by manual inspection prior to the capture of the second image. Images were processed and analyzed using MetaMorph software according to manufacturer’s recommendations (Molecular Devices).

**Mass Spectrometry**

The cleavage sites within Aß40 and Aß42 hydrolyzed by CatD were determined essentially as described [6] with minor modifications.  Briefly, biotinylated Aß peptides were incubated for various lengths of time with purified CatD. The reaction was stopped by addition of PepA (1 µM) and pH adjustment. Aß fragments were precipitated by magnetic beads coated with streptavidin (for biotinylated Aß). Beads were washed with 10 mM NH4CO3, pH 8.0, and peptide fragments were eluted using 0.5% trifluoroacetic acid in 75% acetonitrile in water, followed by the addition of an equal volume of a saturated sinapic acid solution dissolved in 0.5% trifluoroacetic acid in 50% acetonitrile and water. Digested products were spotted onto gold chip and analyzed using a Ciphergen ProteinChip SELDI time-of-flight system (Bio-Rad). Mass spectra were acquired automatically in a linear positive mode at 1350 shots per spectrum.

### Enzymological studies

### For experiments based on radiolabeled Aß peptides, ^125^I-Aß42 and ^125^I-Aß40 prepared as described [7] (generous gift of R. Deane and B. Zlokovic) were mixed in a 1:10 ratio with the corresponding unlabeled Aß peptides, and degradation was quantified by trichloroacetic acid precipitation, as described [8]. Kinetic parameters obtained from “silent substrate” experiments with the fluorogenic substrate were carried out and calculated from the resulting data as described [9]. Homogeneous time-resolved fluorescence (HTRF)-based kinetic experiments were carried out using end-specific anti-Aß42 and -Aß40 antibodies (Wako) labeled with europium cryptate and N-terminal antibodies labeled with XL665 prepared and quantified according to manufacturer’s recommendations on a SpectraMax^®^ M5^e^ multilabel plate reader (Molecular Devices). Fibrils, protofibrils and SDS-induced oligomers of Aß42 were prepared and quantified as described [10].

**Supplemental References**

1. Bertram L, McQueen MB, Mullin K, Blacker D, Tanzi RE. Systematic meta-analyses of Alzheimer disease genetic association studies: the AlzGene database. Nat Genet. 2007;39(1):17-23.

2. Leissring MA, Farris W, Chang AY, Walsh DM, Wu X, Sun X, et al. Enhanced proteolysis of beta-amyloid in APP transgenic mice prevents plaque formation, secondary pathology, and premature death. Neuron. 2003;40(6):1087-93.

3. Vekrellis K, Ye Z, Qiu WQ, Walsh D, Hartley D, Chesneau V, et al. Neurons regulate extracellular levels of amyloid beta-protein via proteolysis by insulin-degrading enzyme. J Neurosci. 2000;20(5):1657-65.

4. Dickson DW, Bergeron C, Chin SS, Duyckaerts C, Horoupian D, Ikeda K, et al. Office of Rare Diseases neuropathologic criteria for corticobasal degeneration. J Neuropathol Exp Neurol. 2002;61(11):935-46.

5. Farris W, Mansourian S, Leissring MA, Eckman EA, Bertram L, Eckman CB, et al. Partial loss-of-function mutations in insulin-degrading enzyme that induce diabetes also impair degradation of amyloid beta-protein. Am J Pathol. 2004;164(4):1425-34.

6. Abdul-Hay SO, Sahara T, McBride M, Kang D, Leissring MA. Identification of BACE2 as an avid ß-amyloid-degrading protease. Mol Neurodegener. 2012;7(1):46.

7. LaRue B, Hogg E, Sagare A, Jovanovic S, Maness L, Maurer C, et al. Method for measurement of the blood-brain barrier permeability in the perfused mouse brain: application to amyloid-beta peptide in wild type and Alzheimer's Tg2576 mice. J Neurosci Methods. 2004;138(1-2):233-42.

8. Leissring MA, Lu A, Condron MM, Teplow DB, Stein RL, Farris W, et al. Kinetics of amyloid beta-protein degradation determined by novel fluorescence- and fluorescence polarization-based assays. J Biol Chem. 2003;278(39):37314-20.

9. Case A, Huskey WP, Stein RL. Enzymatic reaction of silent substrates: kinetic theory and application to the serine protease chymotrypsin. Biochemistry. 2003;42(16):4727-32.

10. Rangachari V, Moore BD, Reed DK, Sonoda LK, Bridges AW, Conboy E, et al. Amyloid-beta(1-42) rapidly forms protofibrils and oligomers by distinct pathways in low concentrations of sodium dodecylsulfate. Biochemistry. 2007;46(43):12451-62.
